# Is Gastroparesis Found More Frequently in Patients with Cystic Fibrosis? A Systematic Review

**DOI:** 10.1155/2016/2918139

**Published:** 2016-05-30

**Authors:** Juan E. Corral, Corey W. Dye, Maria R. Mascarenhas, Jamie S. Barkin, Matthias Salathe, Baharak Moshiree

**Affiliations:** ^1^Department of Internal Medicine, University of Miami Miller School of Medicine, Miami, FL 33136, USA; ^2^Division of Gastroenterology, Hepatology and Nutrition, The Children's Hospital of Philadelphia, Philadelphia, PA 19104, USA; ^3^Division of Gastroenterology, University of Miami Miller School of Medicine, Miami, FL 33136, USA; ^4^Division of Pulmonary, Allergy, Critical Care, and Sleep Medicine, University of Miami Miller School of Medicine, Miami, FL 33136, USA

## Abstract

Cystic fibrosis (CF) is associated with different gastrointestinal motility disturbances and syndromes. We aim to assess gastric emptying in patients with CF compared to healthy controls by a systematic review of existing literature. Medical databases and abstracts from major gastroenterology and CF meetings were reviewed. Emptying times in CF patients were compared with healthy controls using random effects models. Subgroup analysis stratified results by age and diagnostic modality. Nineteen studies from 7 countries included 574 subjects (359 CF patients and 215 controls). Using pooled analysis frequency of gastroparesis was high (38%, 95% CI 30–45%) but results were highly dependent on the diagnostic modality. Delayed gastric emptying is more common in CF compared to general population. Scintigraphy identified rapid gastric emptying in a subgroup of CF patients, but this finding disappeared with adequate pancreatic enzyme replacement and after other diagnostic modalities were included.

## 1. Introduction

Cystic fibrosis (CF) is associated with gastrointestinal dysmotility such as gastroparesis (GP), gastroesophageal reflux, meconium ileus, distal intestinal obstruction syndrome, and chronic constipation [1]. It is well known that small bowel and total transit time are prolonged in CF [1–4]. However, the effects of cystic fibrosis on gastric emptying (GE) and the incidence of GP in this population are variable. Studies report either more rapid GE in CF patients compared to controls, slower GE in CF patients, or no difference between both groups.

The diagnosis of GP has serious implications in CF care as it can worsen the chronic malnutrition associated with the disease due to reduced oral caloric intake and reduce patients' quality of life. GP may also interfere with oral medication delivery and absorption as suggested by studies evaluating pancreatic enzyme replacement therapy (PERT) used in patients with CF and pancreatic insufficiency [5]. About 90% of patients with CF have exocrine pancreatic insufficiency and require regular PERT to improve the digestion of dietary fat, protein, and other nutrients [6]. PERT improves but does not necessarily normalize fat digestion [5, 7, 8]. Differences in response to PERT may be related to gastric emptying rates, as digestion of fat in patients with CF and pancreatic insufficiency is strongly affected by how rapidly fat enters the duodenum [9]. Additionally, macrolides (e.g., azithromycin) are used as a chronic anti-inflammatory therapy in the patients who suffer from* Pseudomonas* infection in their lungs. This therapy has prokinetic effects that may improve GP or cause GI distress due to rapid gastric emptying [10, 11].

This systematic review aims to determine whether patients with CF have slower or faster GE compared to healthy controls. The analysis did not intend to assess whether disorders in gastric emptying were associated with upper gastrointestinal symptoms since symptoms are poor predictors of motility, regardless of the technique used [12].

## 2. Methods 

### 2.1. Search Strategy

We performed a literature search in September 2014 using PUBMED, EMBASE, Web of Science, and Scopus databases. Two authors (Juan E. Corral and Corey W. Dye), conducted the initial screening independently, using the following search terms: (gastroparesis (MeSH term), gastric emptying (MeSH), or gastric scintigraphy (not MeSH term)) and (cystic fibrosis (MeSH)). No language filters were used. We also reviewed the available abstracts in summaries from major gastroenterology meetings (DDW: Digestive Disease Week, ACG: American College of Gastroenterology, and EUG: United European Gastroenterology), and cystic fibrosis conferences (NACFC: North American Cystic Fibrosis Conference and ECFS: European Cystic Fibrosis Society). We then reviewed the reference lists from retrieved articles to identify further relevant studies. Authors were contacted to provide additional information when an e-mail was provided. This systematic review was planned, conducted, and reported in adherence to MOOSE Group recommendations [13].

### 2.2. Eligibility Criteria

Studies were considered in this systematic review if they met the following inclusion criteria: they were performed in human subjects, included an abstract with a methods section, and provided at least one measurement of gastric emptying using either Tc-Scintigraphy, a wireless capsule, or C-Octanoic breath test. Single case reports and review articles were excluded from our sample but all other available studies (case-series, case controls, cohorts, and clinical trials) were considered for initial analysis. A second selection was done within that group to only include studies that compared CF patients with healthy controls. When multiple publications were reported from the same population, the report with the largest sample was selected. Studies that used nonconventional techniques (e.g., ultrasound and fluoroscopy) or that assessed total intestinal time, small bowel, or colon motility but did not have separate measurements of the stomach motility parameters were excluded from analysis. Studies that measured gastric contractions (e.g., electrogastrography) as an indirect measure of gastric emptying were also excluded. Inclusion was not otherwise restricted by language, study size, or setting.

### 2.3. Data Extraction

The following data were extracted from each study: first author's last name, publication year, country where the study was performed, study period, sample size (number of patients with CF and controls), participant's sex and age, diagnostic modality (e.g., scintigraphy and C-Octanoic scan), type of test meal used to deliver marker (solid meal or liquid), available gastric emptying measurements, and number of cases with diabetes mellitus (DM) and with pancreatic insufficiency. We recorded the following GE measurements: total gastric emptying time, gastric emptying half time (*T*_1/2_), and percentage of retention at 1, 2, 3, or 4 hours (RR1, RR2, RR3, and RR4). The number of participants with GP according to original manuscript criteria was also documented.

### 2.4. Statistical Analysis

All studies where the percentage of patients with CF and GP could be estimated were included in the initial review. The percentage of patients with GP and corresponding 95% confidence intervals (95% CIs) were calculated using the Poisson distribution. Forest plot graphs were elaborated using a random effects model selected* a priori*.

Primary analysis pooled all studies that included a control comparison group in a similar fashion. Two studies reported gastric emptying means in case and control groups without their respective dispersion measurements (standard deviation). For those cases, we used standard deviations described in other measurements. For example, when mean and standard deviation was provided for RR1 but not for RR2, we used the standard deviation reported for RR1. Studies that did not report point estimates (mean or median) were not included in the systematic review [21, 22].

For case control studies, methodological quality was assessed by two authors (Juan E. Corral and Baharak Moshiree) using the 9-star Newcastle-Ottawa Scale [30]. Percentage of agreement and bias index were estimated to measure interobserver agreement.

Subgroup analysis was conducted to evaluate for two effect modifying variables: diagnostic modality and age (mean age less or more than 18 years). Statistical heterogeneity between studies was evaluated by calculating *I* ^2^ statistics. Publication bias was evaluated by visual inspection of funnel plots and Egger's bias test. All statistical analyses were performed with Stata version SE 11.2 (Stata-Corp, College Station, TX, USA).

## 3. Results

### 3.1. Literature Search

**Figure 1:**
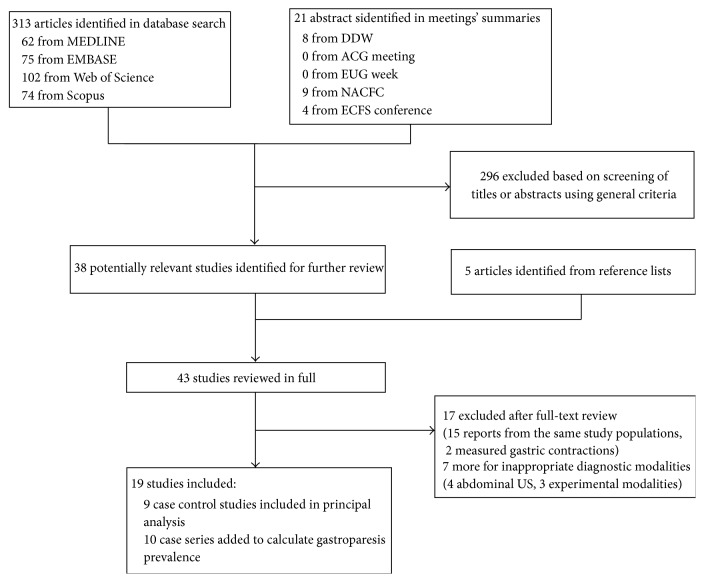
Selection of studies for inclusion in systematic review. DDW: Digestive Disease Week, ACG: American College of Gastroenterology, EUG: United European Gastroenterology, NACFC: North American Cystic Fibrosis Conference, and ECFS: European Cystic Fibrosis Society.

The detailed steps of our literature search are shown in Figure 1. We identified 43 potentially relevant articles concerning gastric emptying in CF. Fifteen articles were excluded because of duplicate reports from the same study population. Two more studies were excluded because they used electrogastrography reporting contractile activity rather than gastric emptying. Nineteen studies were included in the final review, 9 case control studies, and 10 more without a control group.

### 3.2. Study Characteristics

Nineteen studies were published between 1995 and 2013 and included a total of 574 subjects from 9 different countries (359 patients with CF and 215 controls). See Table 1. Mean age of participants ranged from a few months to 28 years.

Eleven studies used Technetium scintigraphy [4, 20–29], 6 used C-Octanoic breath test [5, 9, 16–19], and 2 used wireless motility capsule [14, 15]. The test meal used to deliver marker varied between studies. Two studies used liquids, 14 studies used solid food, and two studies used both.

### 3.3. Frequency of Gastroparesis in Cystic Fibrosis

**Figure 2:**
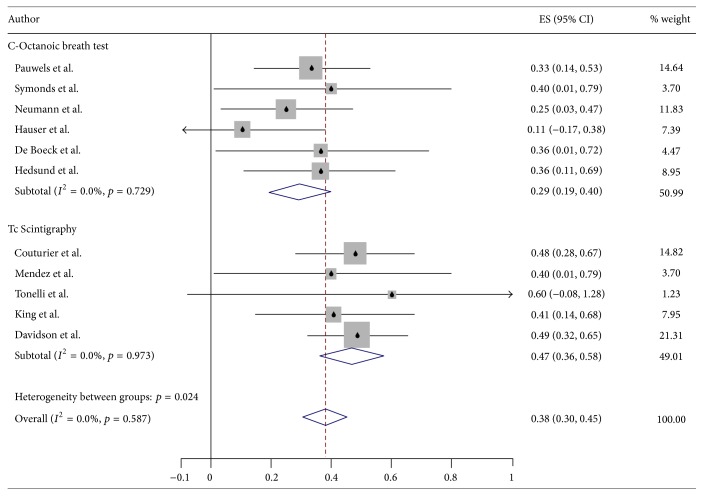
Forest plot graph for frequency of gastroparesis in cystic fibrosis, divided by measuring technique.

From all available studies (*n* = 19), we were able to estimate the percentage of patients with GP in 11 studies (4 studies with a control group and 7 studies without control group). Pooled analysis estimated that 37.9% (95% CI 30.4–45.4%) have GP (Figure 2). Results were highly dependent on the diagnostic modality used. Scintigraphy revealed higher frequency of GP in CF than C-Octanoic test.

Frequency of GP increased with age, 26.6% (95% CI 13.2–39.9%) in studies with patients younger than 18 years compared to 36.3% (20.9–51.7%) seen in older patients (>18 years), although this difference was not statistically significant.

### 3.4. Cystic Fibrosis Patients Compared to Healthy Controls

Overall, 2 studies reported slower GE, 3 reported faster GE, and 4 reported no difference in patients with CF compared to healthy controls. See Table 2. 359 CF patients and 215 controls were included in primary analysis. In the CF group, 51.8% patients were male, 3.1% had DM, and 100.0% had documented exocrine pancreatic insufficiency. In the control group, 55.8% were male, 0% had DM and 4.38% pancreatic insufficiency.

Four Tc-Scintigraphy studies reported RR1 and RR2, 2 wireless motility capsule studies reported RR3, and 3 studies reported *T*_1/2_ using different techniques. No studies reported RR4 which is now the standardized protocol recommended by the National Nuclear Medicine Society and the Neurogastroenterology and Motility Society [31].

Pooled comparison of the 4 scintigraphy studies [4, 21, 22, 28] reporting retention rates (RR) in 1 or 2 hours favored faster gastric emptying in CF (RR1 SMD −1.62 (95% CI −2.16 −1.09) and RR2 SMD −0.96 (95% CI −1.44 −0.47)). No pooled analysis could be performed for wireless motility studies or C-Octanoic tests, due to missing information.

Within the scintigraphy group, 3 studies showed faster gastric emptying and 2 slower gastric emptying in CF patients. See Table 2. One study reported no difference between groups and the remaining studies did not have any comparison group. Of note, Kuo et al. proved that gastric emptying is faster in CF but adequate PERT slowed gastric emptying substantially to a rate comparable to healthy subjects.

Patients with CF in the studies that revealed rapid gastric emptying were younger than patients in other studies using scintigraphy. Sample size was also smaller (16, 36, 11 versus 88, 101) in those studies. See Table 1.

### 3.5. Risk of Bias Assessment

All studies had small samples (range 10–101), and most had methodological or reporting limitations. Mean Newcastle-Ottawa score was 4.5 (range 1–9). Interobserver agreement between the two reviewers was 35.7% and bias index was 0.36. Heterogeneity was significant (*I* ^2^ range 89–94%). A funnel plot was elaborated for studies included in primary analysis and Egger's bias coefficient was 3.58 (95% CI 2.01–5.16).

## 4. Discussion

This systematic review shows that patients with CF have a high frequency of GP, up to 38% (95% CI 30–45%) according to our estimates. The prevalence of GP in general population is probably low (age-adjusted incidence estimated to be 2.4 per 100,000 person-years for men and 9.8 per 100,000 person-years for women) [32–34]. Our estimate in CF is elevated but still less than the prevalence seen in high-risk groups like diabetics with upper gastrointestinal symptoms, of which 50–65% are diagnosed with GP [35–37]. We also found a higher prevalence of GP in CF studies with patient populations older than 18 years.

Gastroparesis is a diverse syndrome that varies by gender, body mass, symptoms, and severity of gastric emptying delay [38]. The mechanisms through which patients with CF develop GP are likely multifactorial and coexist with a subgroup of patients with rapid gastric emptying.

CF is associated with dysmotility disorders including gastroesophageal reflux, distal intestinal obstruction syndrome, and chronic constipation. Studies using knock-out mice show that the decreased intestinal motility is not caused directly by loss of CFTR but rather that it is a consequence of sequential events associated with small intestinal bacterial overgrowth, luminal viscosity, and inflammation [39, 40]. It is unlikely that the above mechanisms cause GP as these derangements develop downstream in the gastrointestinal tract. However, we hypothesize that multiple neurologic reflexes and neurohumoral pathways induce GP in CF like in other patients with chronic constipation. These reflexes include an abnormal persistence of normal feedback mechanisms from the small bowel (prolonged ileal inhibition of gastric emptying), colonic stasis affecting gastric emptying via neural reflexes, and an abnormal circulating gastrointestinal hormone response to standard oral stimulus [41].

In addition to constipation, malnutrition and a lower BMI have been found to predict delayed gastric emptying in the general population and in patients with CF [25]. The exact mechanism is not clear but critical illness generally induces a high inflammatory state that has been associated with severe GP [42]. Chronic use of opiates and anticholinergics, frequently used in CF, can also decrease gastric emptying and intestinal transit time. Finally, DM is the main cause of secondary GP in adults. Diabetes did not seem to be relevant in this systematic review as it was documented in only 3% of our sample. It is important to consider that only one study reported glucose intolerance or postprandial hyperglycemia. These two disorders can significantly alter GES and would have been missed by strict DM criteria [43].

Despite the high prevalence of GP, no significant difference was found when comparing RR1 and RR2 between CF patients and controls. We attribute this to the broad spectrum of study populations and the different test meals used to diagnose GP. Up to now, scintigraphy is recognized as the gold standard method for studying GE [44]. Three scintigraphy reports described GE to be faster in CF than in controls. Patients enrolled in these studies were younger than patients in the other scintigraphy studies (mean age 12.6–25.8 versus 16.7–28). Risk of bias in both groups of studies was similar (5.5, 7, 5 versus 4, 4.5) but the first group had smaller samples (63 versus 189). Of note, Kuo et al. showed that, without PERT, patients with CF have faster gastric emptying after a high-fat/high-carbohydrate meal, compared with healthy subjects. The authors hypothesized that reduced fat digestion causes diminished small intestinal feedback [28].

Scintigraphy results are highly dependent on the technique used (i.e., type of meal and caloric contents, follow-up time, and degree of hyper or hypoglycemia). To address this issue, the American Neurogastroenterology and Motility Society and the Society of Nuclear Medicine recommended standardized testing in 2008 [31]. Of all scintigraphy studies, only 5 conducted after the consensus recommendations were published and none of them were compliant with the recommendations. Our work emphasizes the need to adopt this standardized protocol to increase reliability and credibility of future research [31].

Results from studies using techniques other than scintigraphy should be interpreted with caution. C-Octanoic breath tests require a normal small bowel absorption and pulmonary function. While intestinal absorption can be almost normal with PERT, pulmonary deterioration is the hallmark of CF. Of all the new techniques, capsule devices have shown promising results. A study reported that wireless motility capsule (Smartpill^©^) had a 38% discordance with conventional testing and provided new dysmotility diagnoses in 53% of patients [12]. Both studies using capsule methodologies revealed no difference between CF patients and controls.

Regarding the methods to deliver markers, the classification of solid or liquid is oversimplistic. When a mixed (liquid plus solid) meal is used, GE rate correlates with liquid emptying better than solid emptying [45]. Differential emptying of solids and liquids occur and alterations in the former do not predict alterations in the latter [46]. Only one study using a solid and liquid test meal reported similar GE rates for both solids and liquids [47]. Again, we advocate for standardized testing whether by scintigraphy or wireless motility testing [31].

In this systematic review the risk for selection bias is significant and particularly for the primary analysis. Given the scarcity of complete readings on GE, only 9 out of the 19 studies were considered acceptable quality and included controls. One study included only patients postlung transplant leading to possible overestimation of GP incidence [48]. Transplant recipients are at increased risk of GI disturbances related to vagal nerve damage after surgery. These patients are known to have GP and are at high risk of developing gastric bezoars [49].

We found significant heterogeneity in GE for the reasons mentioned above and also for the time intervals used. Retention rates at 4 hours are significantly more accurate than 1 or 2 hours readings and studies that provided 4-hour measurements after contrast ingestion were limited [50]. Half emptying times (*T*_1/2_) have even less sensitivity compared to RR1 and RR2. In addition to technical limitations on measurements, the majority of studies had low scores in study design and reporting according to the Newcastle-Ottawa score. Publication bias is possible with the funnel plot suggesting paucity of small studies (large standard error) showing a low incidence of GP.

Although subgroup analysis was performed, only two variables could be included in our model (age and diagnostic modality). Our sample size was too small to allow for metaregression and evaluate for other effect modifying variables. Furthermore, we could not assess the effect of medications on gastric emptying. Future studies that explore the effects of other medications other than PERT (in particular opioids, macrolides, and laxatives like polyethylenglycol) and glucose intolerance in addition to DM are suggested.

This study has significant strengths. This is the first systematic review to assess gastric emptying in CF. We have shown a higher frequency of GP in CF and described the general characteristics of CF patients with rapid gastric emptying as essentially younger patients, with pancreatic insufficiency and PERT held while being tested. We advocate use of standardized testing in this population to evaluate gastric emptying. Delayed emptying is likely a multifactorial process, driven by abnormal neurologic reflexes and neurohumoral pathways, and diabetes is unlikely to play a significant role. There is significant heterogeneity with paucity of high quality studies and possibly interrater disagreement even though bias index was mild-moderate [51].

## 5. Conclusion 

Patients with CF have a high frequency of GP. No significant difference was found when comparing patient with CF to healthy controls by using RR1 or RR2, likely secondary to differences in study design, sample selection, and diagnostic technique used. There is a clearly identified group of patients with CF that have rapid gastric emptying seen with scintigraphy, in small studies enrolling young patients with pancreatic insufficiency not taking PERT before testing.

## Figures and Tables

**Table 1:** Studies evaluating gastric emptying in cystic fibrosis.

Author	Year	Location	Subjects			Gender		Age		DM		Pancreatic insufficiency		Intervention	Result
						Male		Mean							
			CF	Controls	Total	CF	Controls	CF	Controls	CF	Controls	CF	Controls		
Capsule methods															
Hedsund et al. [14]	2012	Denmark	10	16	**26**	5	9	23	26	0	0	10	0	No	No difference
Gelfond et al. [15]	2013	Buffalo, NY	10	10	**20**	3	3	21.7	22.5	0	0	10	0	No	No difference

C-Octanoic breath test															
De Boeck et al. [5]	1998	Belgium	11	—	**11**	4	—	10.5	—	—	—	11	—	Creon and high-lipase Creon in CF.	No control group
Symonds et al. [9]	2003	Australia	10	12	**22**	5	7	10.2	12.5	NA	0	10	0	Pancreas in CF	No difference
Neumann et al. [16]	2003	Czech Republic	20	—	**20**	NA	—	13.5	—	—	—	—	—	No	No difference
Hauser et al. [17]	2010	Belgium	19	—	**19**	13	—	5	—	—	—	—	—	No	No control group
Pauwels et al. [18]	2011	Belgium	33	—	**33**	18	—	28	—	0	—	31	—	No	No control group
Hauser et al. [19]	2013	Belgium	22	—	**22**	12	—	9	—	—	—	0	—	No	No control group

Scintigraphy with technetium															
Davidson et al. [20]	1995	Canada	70	—	**70**	NA	—	NA	—	—	—	—	—	No	No control group
Carney et al. [21]	1995	Australia	5	11	**16**	3	4	25	21	0	0	5	0	No	CF Faster
Collins et al. [22]	1997	Australia	19	17	**36**	8	8	12.6	12.8	2	0	19	0	Usual dose of PERT in CF	CF Faster
Munck et al. [23]	1997	France	7	—	**7**	NA	—	NA	—	—	—	—	—	Erythromycin in CF	No control group
Couturier et al. [24]	2004	France	48	53	**101**	NA	42	NA	31.84	NA	0	NA	0	No	CF Slower
King et al. [25]	2006	Australia	22	—	**22**	8	—	NA	—	—	—	18	—	Usual dose of PERT in CF	No control group
Tonelli et al. [26]	2009	Gainesville, FL	5	—	**5**	1	—	25.2	—	2	—	5	—	IV Erythromycin in CF	No control group
Luu et al. [27]	2011	Canada	17	—	**17**	NA	—	NA	—	—	—	—	—	Measurements before and after Cisapride in CF	No control group
Kuo et al. [28]	2011	Australia	5	6	**11**	3	3	25.8	21.7	0	0	5	6	Creon versus Placebo in CF	CF Faster
Mendez et al. [29]	2012	Chicago, Il	10	78	**88**	8	38	28	59	NA	NA	NA	NA	No	CF Slower
Rovner et al. [4]	2013	Philadelphia, PA	16	12	**28**	9	6	16.7	28.6	0	0	16	0	Creon in CF	No difference
			359	215	**574**	100 (50.76%)^a^	120 (55.81%)^a^			4 (5.71%)^a^	0 (0.00%)^a^	140 (84.85%)^a^	6 (4.38%)^a^		

^a^Of available data.

CF: cystic fibrosis and NA: data not available.

**Table 2:** Case-control (higher-quality) studies evaluating gastric emptying in cystic fibrosis.

Author	Sample *n* = 348 (CF/control)	Cystic fibrosis						Controls matching	Food used to deliver marker		PERT before testing	Follow-up time	NOS Rater 1/Rater 2	Conclusion
		Diagnosis	DM	Mean BMI	Pancreatic insufficiency	Pulmonary function	Medications							
Capsule methods														
Hedsund et al. [14]	26 (10/16)	All had severe classes I–III mutations (7 were ΔF508 homozygote)	0 (0%)	22	All cases (confirmed by fecal elastase)	NA	GI medication, antibiotics, and probiotics held >72 h	Unmatched	MTS-1 Capsule fasting	NA	Held	7 h max	3/6	No difference (CF slower, but *p* > 0.05)
Gelfond et al. [15]	20 (10/10)	Sweat Cl > 60 mmol/L or 2 CF mutations (6 were ΔF508 homozygote)	0 (0%)	22.9	All cases (confirmed by fecal elastase, coefficient of fat absorption or serum trypsinogen)	FEV1 > 25% predicted	Antiacids and antibiotics held >1 week	Age, sex, BMI	Smartpill with low fat meal bar	Solid	Half their usual dose	72 h max	5/6	No difference

C-Octanoic breath test														
Symonds et al. [9]	22 (10/12)	Sweat Cl > 80 mmol/L or “genotype determination”	NA	NA	All cases (confirmed by coefficient of fat absorption or steatorrhea + microscopic stool fat)	NA	Not taking any GI motility medication	Unmatched	Pancake, toast, orange juice, and milk	Solid	Usual dose	8 h	3/6	No difference

Scintigraphy with technetium														
Carney et al. [21]	16 (5/11)	NA	0 (0%)	NA	All cases (confirmed by steatorrhea + high 3-day fecal fat)	FEV1 > 50% predicted	NA	Unmatched	Soup and olive oil	Liquid	Held 14 h prior	2 h	4/7	GE of fat is faster in CF
Collins et al. [22]	36 (19/17)	NA	2 (10.5%)	NA	All cases (confirmed by high 3-day fecal fat)	NA	Not taking any GI motility medication >4 days	Age, sex	Pancake	Solid	Usual dose	2 h	7/7	GE is faster in CF
Couturier et al. [24]	101 (48/53)	NA	NA	NA	NA	Severe pulmonary disease pre transplant	NA	Unmatched	Egg, bread meal, and water	Solid	NA	2/3 of stomach emptied	5/4	GE is slower in CF (*p* = 0.001)
Kuo et al. [28]	11 (5/6)	NA	0 (0%) 1 with glucose intolerance	20.0	All cases (NA)	NA	NA	Unmatched	Smashed potatoes	Solid	Crossover Arm 1: no enzymes. Arm 2: twice the usual dose	3 h	4/6	GE is faster in CF (*p* < 0.001). But PERT slowed GE substantially in CF to a rate comparable to healthy subjects
Mendez et al. [29]	88 (10/78)	NA	NA	20	NA	Postlung transplant	NA	Both CF and controls had lung transplant^a^	Egg	Solid	NA	1.5 h	4/4	Both groups had delayed GE, but CF > controls (44% and 29%, resp.)
Rovner et al. [4]	28 (16/12)	NA	0 (0%)	19.6	All cases (confirmed by a fecal elastase)^b^	FEV1 > 50% predicted	NA	Unmatched	Chocolate shake	Liquid	Standard dose (80,000 lipase U)	6 h	6/9	No difference

^a^78 controls had end-stage pulmonary disease: 36, COPD; 32, idiopathic pulmonary fibrosis; 7, alpha-1-antitrypsin deficiency; and 3, scleroderma.

^b^Four subjects with CF had history of distal DIOS and 1 had history of bacterial overgrowth not being treated at the time of study.

GE = gastric emptying.

CF: cystic fibrosis, NOS: Newcastle-Ottawa score, NA: data not available, DM: diabetes mellitus, and DIOS: distal intestinal obstructive syndrome.

## Abbreviations

CF: Cystic fibrosis
DM: Diabetes mellitus
GE: Gastric emptying
GI: Gastrointestinal
GP: Gastroparesis
PERT: Pancreatic enzyme replacement therapy
RR1: Retention rate in 1 hour
RR2: Retention rate in 2 hours
SMD: Standard mean deviation
*T*_1/2_: Half gastric emptying time.
